# Endothelial Cell Loss after Phacoemulsification according to Different Anterior Chamber Depths

**DOI:** 10.1155/2015/210716

**Published:** 2015-08-31

**Authors:** Hyung Bin Hwang, Byul Lyu, Hye Bin Yim, Na Young Lee

**Affiliations:** Department of Ophthalmology, Incheon St. Mary's Hospital, College of Medicine, The Catholic University of Korea, 222 Banpo-daero, Seocho-gu, Seoul 137-701, Republic of Korea

## Abstract

*Purpose*. To compare the loss of corneal endothelial cells after phacoemulsification according to different anterior chamber depths (ACDs). *Methods*. We conducted a prospective study on 135 eyes with senile cataracts. Eyes with nuclear density grades of 2 to 4 were divided into three groups according to ACD: ACD I, 1.5 < ACD ≤ 2.5 mm; ACD II, 2.5 < ACD ≤ 3.5 mm; or ACD III, 3.5 < ACD ≤ 4.5 mm. Intraoperative mean cumulative dissipated energy (CDE) was measured. Clinical examinations included central corneal thickness (CCT) and endothelial cell count (ECC) preoperatively and 2 months postoperatively. *Results*. There were no significant differences in CDE among the ACD groups (*P* > 0.05). Endothelial cell loss was significantly higher in ACD I than in ACD III in grades 3 and 4 cataract density groups 2 months after phacoemulsification (*P* < 0.05). There were also more changes in CCT in all of the cataract density groups in the ACD I group compared to the ACD II and III groups 2 months postoperatively, but the difference was not statistically significant. *Conclusions*. Eyes with shallow ACDs, especially those with relatively hard cataract densities, can be vulnerable to more corneal endothelial cell loss in phacoemulsification surgery.

## 1. Introduction

Corneal endothelial cells are nonreplicative, and the loss of these cells is only compensated for by the migration, enlargement, and increasing heterogeneity of the cells [1]. Loss of endothelial function by the damage of endothelial cells can lead to increased corneal thickness and decreased corneal transparency because of increased stromal hydration due to compromised pump function [2]. Corneal decompensation is a rare but potentially vision-threatening complication after phacoemulsification surgery. Thus, the evaluation of risk factors for preoperative, intraoperative, and postoperative endothelial cell loss provides important information for the cataract surgeon. Some unfavorable preoperative factors and improper intraoperative procedures can lead to corneal decompensation after phacoemulsification surgery. Several studies have reported that some preoperative and intraoperative parameters influence the risk of endothelial cell loss after phacoemulsification. Specifically, advanced age, hard nucleus density, high ultrasound energy, long phacoemulsification time, the phacoemulsification technique, and large infusion volumes can increase the risk of endothelial cell loss after phacoemulsification [3–6].

Phacoemulsification surgery is performed in a limited, confined space; however, securing adequate surgical space during an operation can decrease the risk of corneal endothelial cell loss as a result of the phacoemulsification procedure. Thus, anatomical and surgical factors, such as adequate anterior chamber depth (ACD), are important for preserving these cells from the mechanical and thermal damage that can occur during the procedure. Some studies have demonstrated that ACD did not affect endothelial cell loss after phacoemulsification surgery using a statistical correlation method [6, 7]. However, these studies did not give careful consideration to other surgical factors, such as cumulative dissipated energy (CDE), ultrasound time (UST), and balanced salt solution (BSS) use as confounding factors. It is well known that UST and ultrasound power are important risk factors for endothelial cell loss after phacoemulsification [3]. Thus, we should control for these factors in evaluating the effects of anatomical factors on endothelial cell loss after phacoemulsification. To the best of our knowledge, no stratified controlled study has compared endothelial cell damage according to different ACDs, controlling for confounding factors such as age, cataract nucleus density, CDE, UST, and BSS use. Thus, we compared corneal endothelial cell loss according to different ACDs in patients with various cataract nucleus densities.

## 2. Materials and Methods

The present prospective stratified controlled study examined eyes with cataracts that were randomly assigned to have phacoemulsification and posterior chamber intraocular lens (IOL) implantation at St. Mary's Hospital between May 2012 and March 2015. This project was approved by the Ethics Committees of Incheon St. Mary's Hospital, Incheon, Korea. All of the subjects provided written informed consent before participation. The study conformed to the tenets of the Declaration of Helsinki.

We prospectively examined 135 eyes in 135 patients scheduled to undergo phacoemulsification surgery. Specifically, we divided patients into three groups according to ACD: ACD I, 1.5 < ACD ≤ 2.5 mm; ACD II, 2.5 < ACD ≤ 3.5 mm; and ACD III, 3.5 < ACD ≤ 4.5 mm. Each ACD group was further divided into three subgroups according to three cataract densities (nuclear opalescence [NO]2, NO3, and NO4). Then, we recruited 15 eyes of 15 patients equally per subgroup for a total of 135 eyes of 135 patients. We used the Lens Opacities Classification System (LOCS) III for grading the NO of cataracts preoperatively [8]. Exclusion criteria included a history of previous ocular surgery or inflammation, trauma, corneal pathology, ECC less than 2000 cells/mm^2^, and intraoperative complications, such as posterior capsule rupture and postoperative complications.

### 2.1. ACD Measurement

Preoperatively, ACD (mm) was recorded using partial coherence laser interferometry (Zeiss IOL Master; Carl Zeiss AG, Oberkochen, Germany). The IOL Master uses a slit-based measurement method; it measures from the anterior cornea vertex to the anterior lens vertex in calculating ACD. The mechanism is the same as an ultrasound method. Using built-in facilities and programming the IOL Master, five consecutive ACD measurements were recorded and averaged.

### 2.2. ECC and Central Corneal Thickness

The ECC with central corneal thickness (CCT) was measured using a noncontact specular microscope (Konan Noncon ROBO SP-9000; Konan Medical Corporation, Fair Lawn, NJ, USA) preoperatively and 1 month and 2 months postoperatively. CCT was measured at the central cornea using the built-in Pachy mode of the specular microscope. The center method was used for endothelial cell counting. The specular microscopy system calculated the ECC using a recorded picture of the endothelial cells. While identifying the center of each endothelial cell, the cell density (cells/mm^2^) was computed on the basis of 100 identified cells taken from the picture. Endothelial cell loss was calculated by measuring the percentage decrease in endothelial cell density of the central cornea as follows: endothelial cell loss = (preoperative cell count − postoperative cell count)/(preoperative cell count × 100%). One examiner was blinded to which images belonged in which group. At each visit, three photographs were taken for each eye and averaged.

### 2.3. Surgical Technique

Phacoemulsification was performed by the same surgeon (HBH). Infiniti vision system and 0.9 mm flared 45° ABS Kelman microtip (Alcon Laboratories, Inc., Fort Worth, TX, USA) were used in all of the surgeries. Ozil torsional ultrasound was used, and the torsional amplitude was set at 90% in linear mode. The aspiration flow rate was set at 30 mL/min, and the height of the infusion bottle was set at 90 cm. In all of the cases, a clear corneal incision was made at a temporal corneal site with a 2.85 mm double-beveled incision knife (Diamatrix Ltd., Inc., TX, USA). Then, the ophthalmic viscosurgical device (OVD; Viscoat, Alcon Laboratories, Inc.) was injected into the anterior chamber. A 5 mm continuous curvilinear capsulorhexis was made using the Masket Capsulorhexis Forceps (Katena Inc., Denville, NJ, USA). Hydrodissection and hydrodelineation were performed using a BSS. In all of the cases, the “divide-and-conquer” technique was used for phacoemulsification. That is, four trenches were sculpted, and the nucleus was divided bimanually into four segments, after which the four divided quadrants were emulsified in the capsular bag. Next, 1% sodium hyaluronate (Healon) was injected into the anterior chamber and capsular bag, and a hydrophilic acrylic IOL (Akreos AO MI60; Bausch & Lomb, Rochester, NY, USA) was implanted in the capsular bag. In all of the cases, the IOL was implanted under the protection of an OVD, which was subsequently removed through irrigation and aspiration. The clear corneal wound was sutured with 10–0 nylon only once. After the surgery, 1% prednisolone acetate (Pred Forte, Allergan, Irvine, CA, USA) and 0.3% gatifloxacin (Gatiflo, Handok, Chungbuk, Korea) were applied four times per day for 2 months.

### 2.4. Intraoperative and Postoperative Measurements

Intraoperative measurements included total BSS volume used, UST, and mean CDE. Postoperative parameters, postoperative CCT and ECC and corrected distance visual acuity (CDVA), were measured at 1 day, 1 month, and 2 months.

### 2.5. Statistical Analysis

All of the data are expressed as means ± standard deviation (SD). For comparison of preoperative (age, CDVA, CCT, and ECC), intraoperative (UST, CDE, and BSS use), and postoperative measurements (CDVA) in the three ACD groups in the same nuclear opacity subgroups, one-way analysis of variance (ANOVA) was used. For pairwise comparisons, the Bonferroni and Dunnett's T3 tests were used as post hoc analyses. For CCT increase and ECC decrease in the three ACD groups in the same nuclear opacity subgroups, analysis of covariance (ANCOVA) was used. For the analysis, age, UST, CDE, and BSS use were set as factors of covariates. For pairwise comparison, Bonferroni and Dunnett's T3 methods were also used as post hoc analyses. Statistical analyses were conducted using the SPSS software (ver. 19.0 for Windows; SPSS Inc., Chicago, IL, USA). A *P* value <0.05 was considered statistically significant.

## 3. Results

### 3.1. Overall Characteristics of the Enrolled Patients

In total, 135 eyes of 135 patients were enrolled. Each subgroup of cataract nucleus density included 45 eyes.  Table 1 shows the overall characteristics of the enrolled patients in each ACD group. There was no statistically significant difference in age, mean CDVA, mean CCT, or mean ECC among the groups.

### 3.2. Intraoperative Measurements

Table 2 shows the values of the intraoperative parameters during surgery. BSS use was significantly higher in the NO2 and NO3 groups in the ACD I group than in the ACD II and III groups (*P* < 0.05). However, BSS use showed no statistically significant difference in the NO4 group among the three ACD groups (*P* > 0.05). There was also no statistically significant difference in CDE and UST in the three cataract nucleus densities among the three ACD groups (*P* > 0.05).

### 3.3. CDVA

There was an equal and significant improvement in logMAR CDVA among the three ACD groups, from the preoperative period to 2 months postoperatively, in the NO2, NO3, and NO4 subgroups. However, there was no statistically significant difference in logMAR CDVA at postoperative 2 months in the three cataract nucleus density subgroups among the three ACD groups (*P* > 0.05; Table 3).

### 3.4. CCT

**Figure 1:**
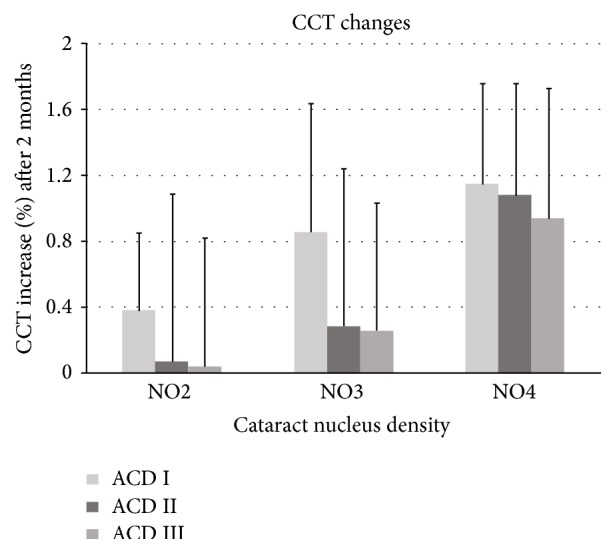
Postoperative changes of CCT by cataract nucleus density and ACD groups (no statistical significance in Bonferroni and Dunnett's T3 as post hoc analysis).

In ANCOVA, setting covariates of age, UST, CDE, and BSS use, although there was less increase in the CCT in all cataract nucleus density subgroups in the ACD III group than the ACD I and II groups 2 months postoperatively, the difference was not statistically significant (*P* > 0.05; Figure 1).

### 3.5. Corneal Endothelial Cell Loss

**Figure 2:**
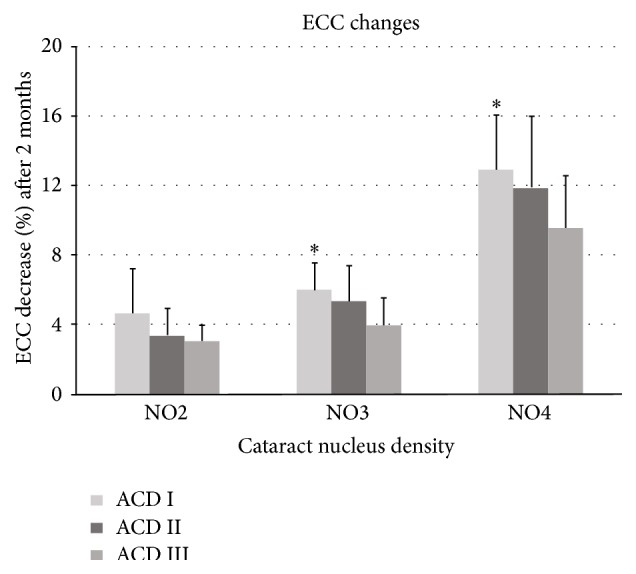
Postoperative endothelial cell loss by cataract nucleus density and ACD groups ( ^*∗*^ = significant in Bonferroni and Dunnett's T3 as post hoc analysis).

In ANCOVA, setting covariates of age, UST, CDE, and BSS use, the mean percentage of endothelial cell loss was significantly different among the three ACD groups in the NO3 and NO4 subgroups (*P* < 0.05). According to the Bonferroni and Dunnett's T3 tests, the mean percentage of endothelial cell loss was significantly higher in the ACD I group (6.04 ± 1.51%; mean ECC, 2658.20 ± 233.04 cells/mm^2^ preoperatively and 2498.60 ± 232.52 cells/mm^2^ 2 months postoperatively) than in the ACD III group (4.01 ± 1.53%; mean ECC, 2602.47 ± 207.51 cells/mm^2^ preoperatively and 2498.93 ± 214.72 cells/mm^2^ 2 months postoperatively) in the NO3 subgroup (*P* < 0.05). In addition, the mean percentage of endothelial cell loss was significantly higher in the ACD I group (12.94 ± 3.16%; mean ECC, 2534.53 ± 272.89 cells/mm^2^ preoperatively and 2206.93 ± 255.44 cells/mm^2^ 2 months postoperatively) than in the ACD III group (9.61 ± 2.96%; mean ECC, 2608.53 ± 298.66 cells/mm^2^ preoperatively and 2359.67 ± 298.91 cells/mm^2^ 2 months postoperatively) in the NO4 subgroup (*P* < 0.05). Although the percentage of endothelial cell loss was higher in the ACD I group than in the ACD II and III groups in the NO2 subgroup, the difference was not statistically significant (ANCOVA, *P* > 0.05; Figure 2).

## 4. Discussion

It is inevitable that endothelial cell damage will occur during the phacoemulsification procedure. Many factors for postoperative endothelial cell loss have been evaluated after phacoemulsification, including cataract density, surgery time, phacoemulsification time, and ultrasound power. In addition, IOL contact, instrument-related trauma, incision size, irrigation solution turbulence, type of IOL, and type of OVD can influence corneal endothelial cell loss after phacoemulsification procedures [9–14]. Corneal endothelial cells are not regenerated once they are damaged. Reuschel et al. [15] found a median postoperative endothelial cell loss of 6.9% (4.5–7.9%) 3 months after cataract surgery.

Some studies already examined and proved that ECC decreases with normal aging process [16–19]. A Portuguese study estimated that ECC decreased 5-6% every 10 years [16]. Møller-Pedersen [17] reported 0.3% reduction of ECC every year and Niederer et al. [18] demonstrated 0.5% reduction every year. And Cheng et al. [19] found annual ECC loss reaching even 1%. Because ECC is negatively correlated to increase of age, we controlled the age factor in evaluating the effects of ACD on ECC loss after phacoemulsification in this study. However, phacoemulsification surgery is known to decrease ECC even more. Reuschel et al. [15] reported ECC loss of 4.5–7.9% 3 months after phacoemulsification. Park et al. [20] demonstrated ECC loss of 5.2–9.1% 2 months after phacoemulsification. These values are similar to our results (4.01–12.94%). At 12 months of followup, Storr-Paulsen et al. [5] reported ECC loss of 3.5–5.7% after the phacoemulsification. In this respect, ECC loss seems to continue at least for a year after cataract surgery and would be larger than that of normal aging process. Further study should be done with longer follow-up period considering the age factor.

Corneal endothelial cell damage can induce corneal decompensation after phacoemulsification, especially in high-risk groups. Thus, endothelial cell loss is an important prognostic factor of the outcome of phacoemulsification surgery, and as such it is important to determine the risk factors of corneal endothelial cell loss, including preoperative, intraoperative, and postoperative parameters, for evaluating the prognosis after surgery. Moreover, increased attention is needed during surgery for high-risk groups.

The phacoemulsification surgery is performed in a limited, confined space; however, adequate space during surgery can decrease the risk of corneal endothelial cell damage by the procedure. Thus, an adequate surgical space is important for decreasing endothelial cell damage from the aforementioned risk factors. Within a shallow ACD, surgery can take place closer to the corneal endothelium. Therefore, we hypothesized that a deep ACD would correlate with lower endothelial cell loss during phacoemulsification surgery.

Advanced age, hard nucleus density, high ultrasound energy, long phacoemulsification time, and large infusion volume can increase the risk of endothelial cell loss after phacoemulsification [3, 4, 6]. Thus, we designated age, UST, CDE, and BSS use as confounding factors (covariates) for evaluating the effect of ACD on endothelial cell loss in phacoemulsification and used ANCOVA as a statistics technique. Previous studies used a statistical correlation method to determine that there is no significant relationship between ACD and endothelial cell loss [6, 7]. However, these studies did not control for other factors, such as age, UST, CDE, and BSS use. In contrast, the present study used a stratified and controlled examination, which increases confidence in the results.

The phacoemulsification technique itself can also influence endothelial cell loss. Storr-Paulsen et al. [5] suggested that the divide-and-conquer technique provokes more endothelial cell loss than the phaco chop technique, because the divide-and-conquer method uses more phaco energy, as it cracks the nucleus and facilitates phacoemulsification. Thus, this technique is more suitable for evaluating the effects of ACD on endothelial cell loss.

Moreover, the phaco platform we used has only Ozil torsional mode in order to exclude the effect of conventional longitudinal phaco energy on ECC loss. Also, CDE was calculated based solely on torsional amplitude and torsional time. However, Reuschel et al. [15] demonstrated that there is no significant difference in ECC loss between groups that used torsional phaco and longitudinal phaco. Similarly, Kim et al. [21] reported that torsional phaco showed no significant difference in ECC loss compared to that of longitudinal phaco at postoperative 1 month.

We used the IOL Master to measure ACD. The IOL Master measures ACD using lateral slit illumination of the cornea and crystalline lens, according to the Scheimpflug principle, and a white light-emitting diode (590 nm) as the light source. The lateral slit illumination is 0.7 mm wide and is used at an angle of 30° during ACD measurements. The axial resolution is precisely 10 *μ*m for ACD measurements. The built-in software measures the distance between the anterior corneal surface and the anterior crystalline lens surface. The IOL Master shows good precision and resolution and is highly repeatable and reliable in measuring ACD compared to the other devices and techniques, such as Visante optical coherence tomography (OCT), slit lamp OCT, Pentacam, and Orbscan IIz [22–26].

In the present study, we compared three ACD groups according to nuclear cataract density. We found that shallow ACD could be a risk factor for increasing endothelial cell loss during phacoemulsification. As such, the percentage of corneal endothelial cell loss was higher in the ACD I group than in the ACD III group in eyes with NO3 and NO4 nuclear densities (*P* < 0.05). However, we found no significant difference in postoperative measurements, such as CCT and CDVA, among the three ACD groups in all of the nuclear density subgroups.

There have been some conflicting reports describing the relationship between ACD and endothelial cell loss in phacoemulsification. McCarey et al. [27] demonstrated that surgical instruments could induce more endothelial cell damage, especially in eyes with shallow ACDs. Walkow et al. [6] showed that short axial length could be a risk factor for endothelial cell loss during phacoemulsification, because small confined surgical space in short eyes increases the risk of endothelial touch by surgical instruments and lens fragments. However, the authors could not demonstrate a relationship between ACD and endothelial cell loss. However, O'Brien et al. [3] demonstrated that there was no relationship between ACD or axial length and endothelial cell loss during phacoemulsification, because an adequate surgical space could be obtained using irrigation flow during the operation. In addition, Reuschel et al. [7] reported that ACD was not a risk factor for postoperative endothelial cell loss in their correlation analysis. Jung et al. [28] compared eyes with nanophthalmos and relative anterior microphthalmos with a normal control group in phacoemulsification surgery. They found higher endothelial cell loss of 14.22 ± 18.45% in nanophthalmic eyes (mean ACD, 1.82 ± 0.31 mm) compared to an ECL of 11.57 ± 11.34% within relative anterior microphthalmic eyes (ACD, 1.87 ± 0.24 mm) and an endothelial cell loss of 7.61 ± 8.77% in their normal control eyes (mean, ACD 2.70 ± 1.31 mm). However, their results were not statistically significant. We hypothesized that a shallow ACD would lead to phacoemulsification being performed closer to the endothelium, so that the corneal endothelium could be vulnerable to torsional ultrasound energy, heat energy, movement of lens fragments, and touch by surgical instruments. For this reason, eyes with shallow ACDs are thought to suffer more endothelial cell loss than eyes with deep ACDs.

Our study had some limitations. The enrolled patients were only followed for 2 months. Thus, a long-term study is needed. In addition, our study could not have a blinded design, so other studies with this design are needed. Furthermore, more patients should be enrolled in future studies.

A significant strength of the study is its design, as to the best of our knowledge, this is the first controlled and stratified study to describe the relationship between ACD and corneal endothelial cell loss after phacoemulsification. We demonstrated that a shallow ACD is related to endothelial cell loss in phacoemulsification, especially in patients with relatively hard cataract nuclear densities. Thus, cataract surgeons should pay particular attention to patients with hard cataract nuclear densities and shallow ACDs during phacoemulsification surgery.

## Figures and Tables

**Table 1:** Preoperative clinical characteristics of enrolled patients.

Parameter	ACD I			ACD II			ACD III		
	NO2	NO3	NO4	NO2	NO3	NO4	NO2	NO3	NO4
Mean age (y)	68.60 ± 8.63	70.93 ± 8.46	69.00 ± 8.86	68.73 ± 8.90	71.47 ± 10.25	68.27 ± 9.18	71.67 ± 8.34	69.27 ± 9.35	70.13 ± 7.54
Mean CDVA (logMAR)	0.53 ± 0.15	0.51 ± 0.12	0.56 ± 0.14	0.55 ± 0.17	0.49 ± 0.14	0.52 ± 0.09	0.51 ± 0.15	0.46 ± 0.10	0.52 ± 0.09
Mean CCT (um)	538.00 ± 31.04	551.73 ± 28.28	561.53 ± 31.52	544.13 ± 29.07	543.73 ± 26.87	546.13 ± 28.17	540.73 ± 28.77	540.60 ± 15.27	552.20 ± 24.35
Mean ECC (cells/mm^2^)	2608.13 ± 270.18	2658.20 ± 233.04	2534.53 ± 272.89	2700.67 ± 269.16	2646.27 ± 245.07	2532.53 ± 251.90	2710.73 ± 266.66	2602.47 ± 207.51	2608.53 ± 298.66
*P* value^*∗*^	>0.05	>0.05	>0.05	>0.05	>0.05	>0.05	>0.05	>0.05	>0.05

Means ± SD.

CDVA = corrected distance visual acuity; CCT = central corneal thickness; ECC = endothelial cell count; NO = nucleus opalescence.

^*∗*^Comparison of three ACD groups in the same cataract nuclear opacity subgroups (Bonferroni and Dunnett's T3 as post hoc analysis).

**Table 2:** Comparison of UST, CDE, and BSS use among three ACD groups according to cataract nucleus density.

Parameter	Mean ± SD								
	ACD I			ACD II			ACD III		
	NO2	NO3	NO4	NO2	NO3	NO4	NO2	NO3	NO4
UST (s)	44.44 ± 8.15	52.75 ± 8.25	72.70 ± 19.83	44.81 ± 7.91	54.36 ± 12.81	86.01 ± 26.41	39.11 ± 6.50	52.65 ± 9.33	86.83 ± 20.97
CDE	7.99 ± 1.80	10.16 ± 2.18	16.16 ± 5.95	8.06 ± 1.66	10.56 ± 3.10	18.59 ± 6.08	7.87 ± 1.62	9.21 ± 1.13	17.20 ± 5.08
BSS use (mL)	75.03 ± 6.05^*∗*^	79.74 ± 3.50^*∗*^	94.29 ± 5.84	67.18 ± 8.68	70.65 ± 6.71	87.77 ± 10.27	70.38 ± 9.89	73.95 ± 10.87	93.17 ± 10.28

UST = ultrasound time; CDE = cumulative dissipated energy; NO = nuclear opalescence.

^*∗*^ *P* < 0.05.

**Table 3:** Preoperative and postoperative logMAR CDVA.

Exam	Mean CDVA ± SD								
	ACD I			ACD II			ACD III		
	NO2	NO3	NO4	NO2	NO3	NO4	NO2	NO3	NO4
Preoperatively	0.53 ± 0.15	0.51 ± 0.12	0.56 ± 0.14	0.55 ± 0.17	0.49 ± 0.14	0.52 ± 0.09	0.51 ± 0.15	0.46 ± 0.10	0.52 ± 0.09
1 d postoperatively	0.19 ± 0.07	0.18 ± 0.07	0.16 ± 0.07	0.21 ± 0.06	0.17 ± 0.07	0.18 ± 0.09	0.19 ± 0.06	0.17 ± 0.07	0.19 ± 0.07
1 mo postoperatively	0.05 ± 0.05	0.07 ± 0.06	0.07 ± 0.08	0.05 ± 0.06	0.09 ± 0.05	0.07 ± 0.07	0.07 ± 0.06	0.04 ± 0.06	0.05 ± 0.06
2 mo postoperatively	0.03 ± 0.05	0.05 ± 0.05	0.05 ± 0.06	0.05 ± 0.05	0.03 ± 0.05	0.06 ± 0.06	0.07 ± 0.06	0.03 ± 0.05	0.04 ± 0.06
*P* value^*∗*^	>0.05	>0.05	>0.05	>0.05	>0.05	>0.05	>0.05	>0.05	>0.05

CDVA = corrected distance visual acuity; NO = nuclear opalescence.

^*∗*^Comparison of three ACD groups in the same cataract nuclear opacity subgroups (Bonferroni and Dunnett's T3 as post hoc analysis).
